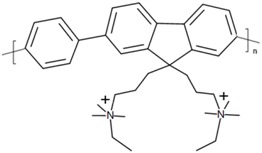# Bulk Heterojunction Solar Cells Based on Blends of Conjugated Polymers with II–VI and IV–VI Inorganic Semiconductor Quantum Dots

**DOI:** 10.3390/polym9020035

**Published:** 2017-01-26

**Authors:** Ryan Kisslinger, Weidi Hua, Karthik Shankar

**Affiliations:** 1 Department of Electrical and Computer Engineering, University of Alberta, 9211-116 St., Edmonton, AB T6G 1H9, Canada; kissling@ualberta.ca (R.K.); weidi@ualberta.ca (W.H.); 2 National Research Council Canada National Institute for Nanotechnology, 11421 Saskatchewan Drive NW, Edmonton, AB T6G 2M9, Canada

**Keywords:** anisotropic nanocrystals, hybrid photovoltaics, interface engineering, nanotechnology, materials processing, tailored nanocomposites, bicontinuous percolation networks, blend characterization

## Abstract

Bulk heterojunction solar cells based on blends of quantum dots and conjugated polymers are a promising configuration for obtaining high-efficiency, cheaply fabricated solution-processed photovoltaic devices. Such devices are of significant interest as they have the potential to leverage the advantages of both types of materials, such as the high mobility, band gap tunability and possibility of multiple exciton generation in quantum dots together with the high mechanical flexibility and large molar extinction coefficient of conjugated polymers. Despite these advantages, the power conversion efficiency (PCE) of these hybrid devices has remained relatively low at around 6%, well behind that of all-organic or all-inorganic solar cells. This is attributed to major challenges that still need to be overcome before conjugated polymer–quantum dot blends can be considered viable for commercial application, such as controlling the film morphology and interfacial structure to ensure efficient charge transfer and charge transport. In this work, we present our findings with respect to the recent development of bulk heterojunctions made from conjugated polymer–quantum dot blends, list the ongoing strategies being attempted to improve performance, and highlight the key areas of research that need to be pursued to further develop this technology.

## 1. Introduction

In society today, fossil fuels such as natural gas, oil and coal dominate the energy market. However, growing concerns over the limited reserves of such fuels and environmental issues has led to heightened interest in the search for alternative energy sources. Solar energy from the sun is by far the largest exploitable energy source, with more energy striking the Earth’s surface in one hour than is consumed by humans in an entire year [1]. The need for cheap, high-performance solar cells is of great importance for the world to be able to meet its growing energy demands. Most solar cells fabricated and in use today are still based on so-called “first generation” technologies. These solar cells, mainly based on silicon wafers, are expensive to produce and inefficient, with relatively little potential for further cost reduction [2]. The second generation of solar cells—fabricated using thin films of semiconducting material such as CdTe and CIGS—decreased fabrication costs and demonstrated interesting properties such as flexibility [3]. However, the significant energy costs associated with their production, including the need for vacuum processes, high temperature and rare elements, has hindered their widespread adoption. Third-generation solar cells have been the focus of intense research over the last decade, and the focus on this area has increased dramatically in only the last few years [4]. Such solar cells comprise a wide variety of technologies including semiconducting conjugated organic materials, quantum dot solar cells and perovskite thin films [5]. These solar cells have seen some commercial adoption for niche markets; however, for them to compete in the larger power market, improvements to their efficiencies and long-term stability are still needed.

The bulk heterojunction (BHJ) solar cell is a third-generation solar cell that in its simplest configuration is comprised of two kinds of material, where one functions as an electron donor and the other functions as an electron acceptor. The working mechanism can be summarized as follows: excitons created by incident photons will form in the donor material and migrate toward the interface between the donor and acceptor material. The acceptor material, having a higher electron affinity with respect to the donor material, will accept the electron from the donor, causing exciton dissociation at the interface. The dissociation of the photogenerated exciton (usually a Frenkel exciton in polymers and small molecules) is itself typically a two-step process consisting of the formation of a charge transfer exciton followed by further dissociation into mobile electron- and hole-polarons [6,7]. The subsequently separated hole polarons and electron polarons will travel through the donor and acceptor materials, respectively, towards their individual electrodes—anode for the holes and cathode for the electrons. The two materials can be simply put into contact (a so-called bilayer heterojunction solar cell) or synthesized as a blend. In a bilayer heterojunction solar cell, geminate recombination due to the small exciton diffusion length in organic semiconductors (typically <15 nm) is a major loss mechanism [8]. In this context, the advantage of a blend (as in a bulk heterojunction) is a larger interface area and an interpenetrating donor–acceptor morphology to minimize the distance that excitons must travel to reach an interface for dissociation. The bulk heterojunction blend also needs to have percolation pathways for both electrons and holes to avoid efficiency losses due to poor charge collection, as will be explained later. The dominant configuration in the field of bulk heterojunction solar cells has consisted of a π-conjugated semiconducting polymer (termed just “polymer” in much of the remainder of this report) as the donor and the small molecule methanofullerene as the acceptor, where efficiencies have reached 11.7% for single junction lab-scale solar cells and 7%–9% for modules [9,10]. The principal disadvantages of the methanofullerene-conjugated polymer bulk heterojunction are the low ambient stability of the blend [11,12], the very low contribution of the fullerene to charge generation, the high exciton binding energy in the polymer—potentially resulting in open circuit photovoltage losses [13]—and the low charge carrier mobilities in both the fullerene (~1 cm^2^·V^−1^·s^−1^) and the polymer (typically 10^−4^−10^−1^ cm^2^·V^−1^·s^−1^) [14]. An alternative configuration is the use of II–VI inorganic semiconductor quantum dots (QDs) and conjugated polymers as the acceptor and donor, respectively. This configuration presents the attractive feature that both components (the QD and the polymer) can contribute to charge generation and potentially complement each other’s spectral absorption ranges to achieve a more complete match to the solar spectrum; there is also the potential for higher charge mobilities (as high as 50 cm^2^·V^−1^·s^−1^) [15] in quantum dot solids and superior ambient stability. While the binding energy of Frenkel excitons in polymers is high (0.2–1 eV) [16,17], the binding energies of Mott–Wannier excitons in the II–VI and IV–VI QDs are typically smaller and more tunable [18].

QDs are semiconductor nanocrystals smaller than the exciton Bohr radius so that the effects of quantum confinement are observed. II–VI semiconductor QDs are primarily made up of elements from Groups 12 and 16 in the periodic table and include compounds such as CdS, CdSe, CdTe, ZnS, ZnO and ZnSe. IV–VI semiconductor QDs are made of elements from Groups 14 and 16 in the periodic table and include compounds such as SnS, PbS and PbSe. Our review is restricted to compounds that strongly absorb at visible and/or near-infrared (NIR) wavelengths. Some advantages of QDs include their size-tuneable electronic bandgap, possibility for multiple exciton generation, high electron affinity, and high electron mobility. They are more commonly used as the acceptor material (i.e., as the *n*-type component) in BHJ solar cells. Quantum dots may be synthesized in a range of sizes, which will in turn affect the size of the QD band gap. Thus, QDs may be tuned to be able to absorb light of different wavelengths. There is also the possibility of harvesting different parts of the solar spectrum by adding different sizes of monodisperse quantum dots (size variation within around 5% of average) into the cell, known as a “rainbow solar cell” which is able to have electronic transitions at different energy levels [19]. Finally, we note that parameters relevant to the efficiency of the bulk heterojunction solar cell such as the size of bound excitons and the charge carrier mobility are positively correlated with the relative permittivity of the blended film, due to which improved performance is expected in QD–polymer blends owing to the increased local dielectric constant resulting from the high permittivity of II–VI semiconductor nanocrystals [20].

While BHJ solar cells incorporating QDs and conjugated polymers may provide significant advantages for the purpose of increased efficiencies—including the tunable band gaps, possibility of multiple exciton generation and photochemical stability of quantum dots with the large molar extinction coefficient and high mechanical flexibility of conjugated polymers—reported device energy conversion efficiencies seem to have plateaued at ~3%–5% using a variety of quantum dots (CdSe, PbS, PbSe, CdTe) and different conjugated polymers (polyphenylenevinylenes, polythiophenes and copolymers of fluorenes, carbazoles, benzothiazadoles, diketopyrrolopyrroles, etc.) [21,22,23,24,25,26,27,28,29,30]. Therefore, significant improvements are needed, especially with regards to charge separation and transport. For this reason, factors such as film morphology are of great importance to increase their viability for commercial applications as well as a deeper fundamental understanding of the mechanisms involved when the two material classes of II–VI quantum dots and conjugated polymers are used together.

## 2. Operation of a Hybrid Solar Cell

### 2.1. Overall Energy Conversion

**Figure 1:**
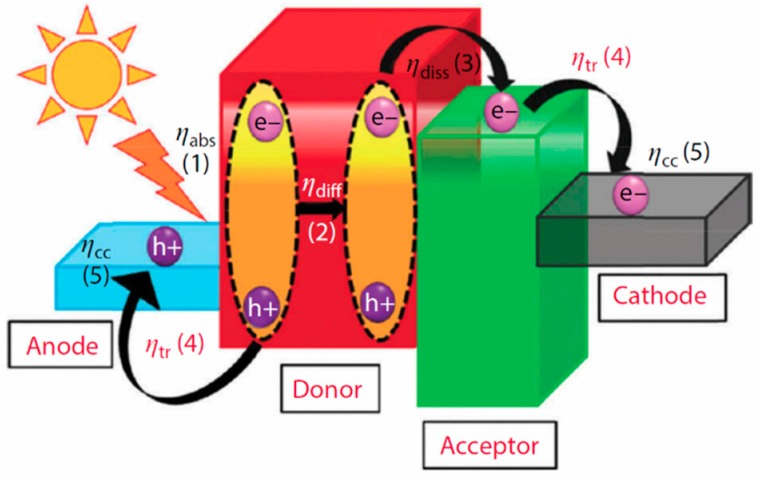
The overall energy arrangement of the conjugated polymer and QD BHJ solar cell, with indication of charge transport and efficiency at each part of the cell. Reproduced with permission from [4]. Copyright 2015 Wiley and Sons.

In the vast majority of photovoltaic bulk heterojunctions, a conjugated polymer is the donor (*p*-type component) due to the scarcity of *n*-type polymer acceptors with good stability, suitable energy levels and acceptable electron mobilities. Small molecule methanofullerenes PC_61_BM and PC_71_BM are most commonly used as the acceptor components in all-organic binary blends. A key difference between a methanofullerene–polymer blend bulk heterojunction solar cell and a semiconductor nanocrystal-polymer blend bulk heterojunction hybrid solar cell is that the polymer is not solely responsible for most of the light absorption since the quantum dots are strong absorbers as well [31]. In contrast, methanofullerenes have a very weak visible light absorption due to the low oscillator strength of electronic transition from the ground state to their first excited state, which is a triplet state [32]. A more subtle difference relates to mutual solubility of the blend components; it has been established that the significant solubility of PCBM in the polymer phase greatly facilitates carrier transport following charge separation [33]. This benefit is not present in hybrid solar cells since inorganic QDs are insoluble in the organic phase. As shown in Figure 1, the donor has a higher lowest unoccupied molecular orbital (LUMO) than the conduction band minimum of the acceptor quantum dot, followed on the right side of the figure by a cathode made of a metal whose Fermi level is slightly lower or roughly proximate with the conduction band of the quantum dot. Thus, the electron flows from the donor towards the cathode. The valence band maximum of the acceptor is the lowest energy level; the next lowest is the donor’s highest occupied molecular orbital (HOMO). On the left side of the energy diagram in Figure 1, a material that has a Fermi level roughly proximate or slightly higher than the HOMO of the donor material is chosen as the anode. As displayed in the diagram, such a cascaded energy level arrangement will direct the flow of charge through the cell in a manner conducive to charge collection at the electrodes, in turn enabling electricity generation in the external circuit.

**Figure 2:**
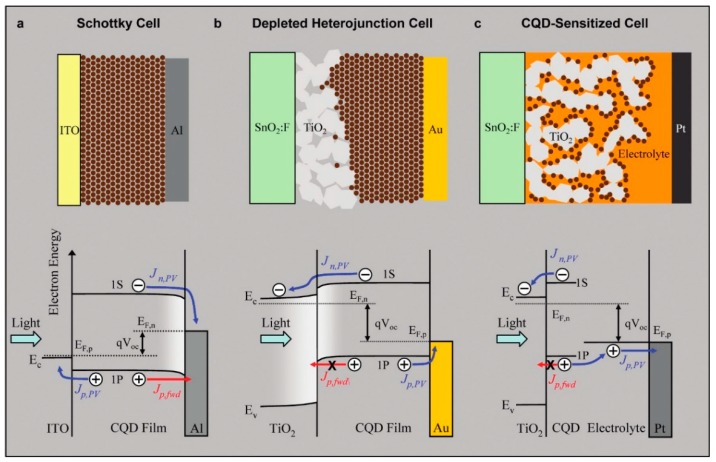
Comparison of three CQD photovoltaic architectures under photovoltaic operation close to maximum *V*_oc_. (**a**) The Schottky design has lower *FF* and *V*_oc_ for a given *J*_sc_, due to the poor barrier for hole injection into the electron-extracting contact; (**b**) the depleted heterojunction design combines the advantages of the other two cells, leading to simultaneously maximized *FF*, *V*_oc_, and *J*_sc_; (**c**) the CQD sensitized cell employs a thin layer of absorber on a high surface area electrode. The light absorbing capacity of this design is lower, leading to poor *J*_sc_, while it provides good *FF* and *V*_oc_. *E*_F,n_ and *E*_F,p_ are the electron and hole quasi-Fermi levels; *E_c_* and *E*_v_ are the conduction and valence band edges; *J_p_*_,PV_ and *J_n,PV_* are the hole and electron photocurrents (and are equal at steady state); *J_p,fwd_* is the hole current in the forward bias direction. Reprinted with permission from [35]. Copyright 2013 American Chemical Society.

The bulk heterojunction configuration is different from the other types of solar cell configurations II–VI and IV–VI semiconductor QDs have been used in, the important ones being listed in Figure 2. The Schottky solar cell in Figure 2a does not require a blend or a second semiconductor component; it instead uses quantum dot solids that act as conduits for both photogenerated electrons and holes—moving in opposite directions toward the cathode and anode, respectively—under the influence of the Schottky junction’s built-in electric field. The depleted heterojunction solar cell in Figure 2b does require a second semiconductor, but the purpose of this layer is principally to create a single-sided p-n junction and fully deplete the film of quantum dot solids of charge carriers at equilibrium. The built-in electric field of the depletion region provides the driving force for charge separation and transport in the QD layer. In Figure 2c, a colloidal quantum dot (CQD)-sensitized photoelectrochemical cell is shown, wherein the quantum dots decorate an n-type nanostructured semiconductor scaffold—typically TiO_2_—and act purely as light absorbers. Charge separation occurs at the TiO_2_–CQD and CQD–electrolyte interfaces, while electron and hole transport occur in the scaffold and electrolyte phases, respectively. The distinguishing feature of the CQD-sensitized photoelectrochemical solar cell is the non-participation of the QDs in charge transport processes. An understanding of these configurations is useful because certain types of bulk heterojunctions incorporate elements from them. For example, Ginger et al. observed that a binary blend of large PbS quantum dots and polythiophene behave somewhat like a conjugated polymer–cathode Schottky solar cell, sensitized by Förster-type resonance energy transfer from the quantum dots to the polymer [34].

**Figure 3:**
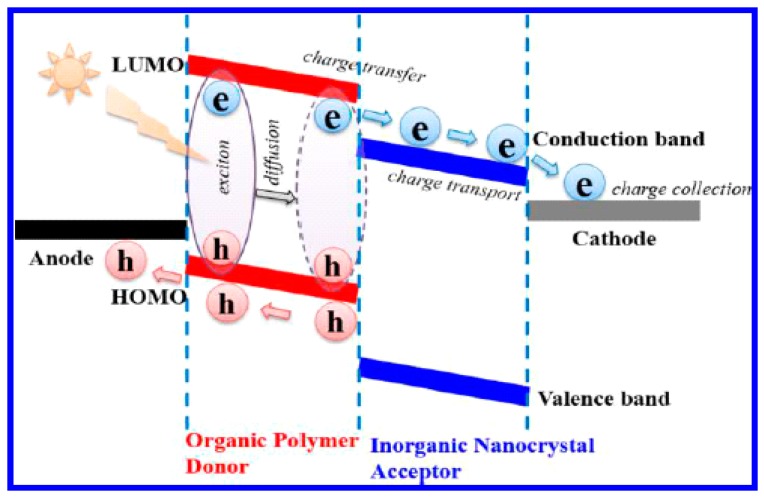
Schematic of the working of inorganic–organic solar cells. An incident photon causes the formation of an exciton in the polymer donor, which dissociates to an electron and hole. The hole flows through the polymer to be collected at the anode, while the electron is transferred to the nanocrystal and collected at the cathode. Reprinted with permission from [36]. Copyright 2013 American Chemical Society.

The quantum yield for sunlight-to-electricity conversion of a hybrid cell can be broken down into five parts, as shown in Figure 3, which are (from left to right) η_abs_, η_diff_, η_diss_, η_tr_ and η_cc_, which correspond to the yield for optical absorption (to create a bound exciton), diffusion of the exciton to an interface, dissociation of the exciton into mobile charge carriers, transport of the charge carriers through the film, and collection of charge carriers by the electrodes, respectively. The total external quantum yield can therefore be expressed as

η_total_ = η_abs_ × η_cc_ × η_tr_ × η_diff_ × η_diss_. (1)

Equation (1) provides a useful framework in which the factors limiting the performance of hybrid solar cells can be understood to make improvements in solar cell design and materials processing. The power conversion efficiency (PCE) of solar cells is given by

PCE = (*J*_sc_ × *V*_oc_ × *FF*)/*P*_in_, (2)

where *J*_sc_ is the short circuit current density, *V*_oc_ is the open circuit photovoltage, *P*_in_ is the incident optical power and *FF* is the fill factor, a measure of the maximum power that can be drawn from the cell.

### 2.2. Equivalent Circuit for Hybrid BHJ Solar Cells

**Figure 4:**
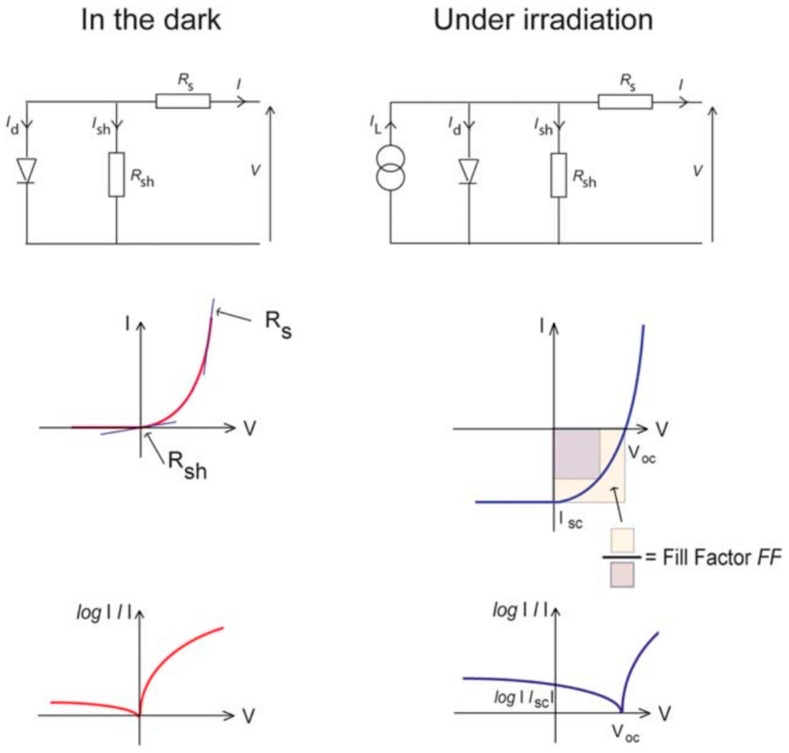
Equivalent circuit diagrams and current-voltage characteristics (in linear and semi-logarithmic scales) for hybrid solar cells in the dark and under irradiation. The definition of fill factor is also shown, as the area of the orange square divided by the area of the purple square. The fill factor, along with *V*_oc_ and *I*_sc_, describes the maximum power from the solar cell. Reproduced from [37] with permission of the Royal Society of Chemistry.

Equivalent circuit diagrams can be modelled for hybrid BHJ devices as shown in Figure 4. The entire device can be modelled as a diode in parallel with a shunt resistor *R*_sh_ (an unwanted path for current between the front and back contacts) and in series with the series resistance *R*_s_ (resistance to the movement of charges through the device and at the contacts). These parameters can be measured by means of dark I–V characterization from the slope of the I–V curve at zero bias and at high forward bias, respectively. When the device is irradiated, a current source, *I*_L_ is added to the equivalent circuit diagram to express the current generating capacity of the solar cell. When the terminals of the hybrid solar cell are shorted, the current measured between these terminals is *I*_sc_, which is typically normalized by the cell area to obtain *J*_sc_. These parameters, along with the irradiated I–V characteristics of the cell, can be used to describe the fill factor (*FF*), which is shown schematically in Figure 4.

### 2.3. Photon Absorption and Formation of Excitons

**Figure 5:**
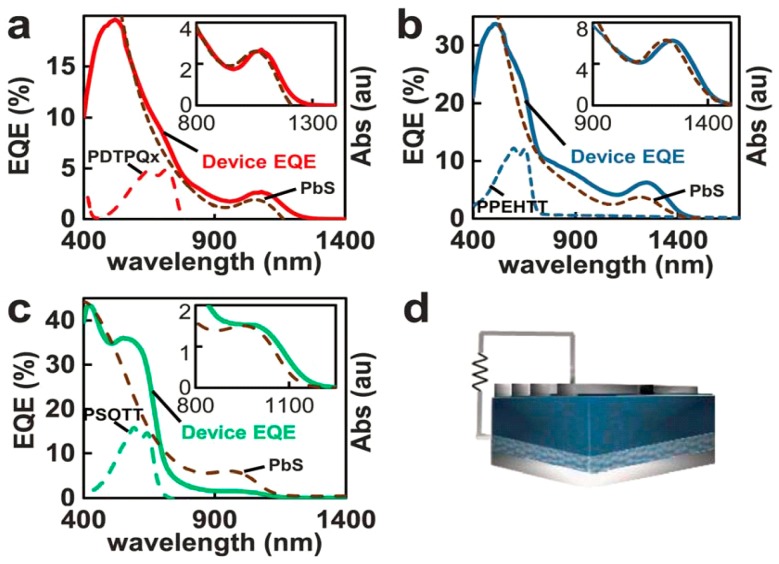
(**a**–**c**) Absorbance spectra (dashed line) of different conjugated polymers and PbS QDs used in the blend, and their corresponding EQE. Insets are a magnified view of the IR region of the spectra. (**d**) Schematic of the design of a QD/polymer hybrid device. Reproduced from [34] with permission of the Royal Society of Chemistry.

**Figure 6:**
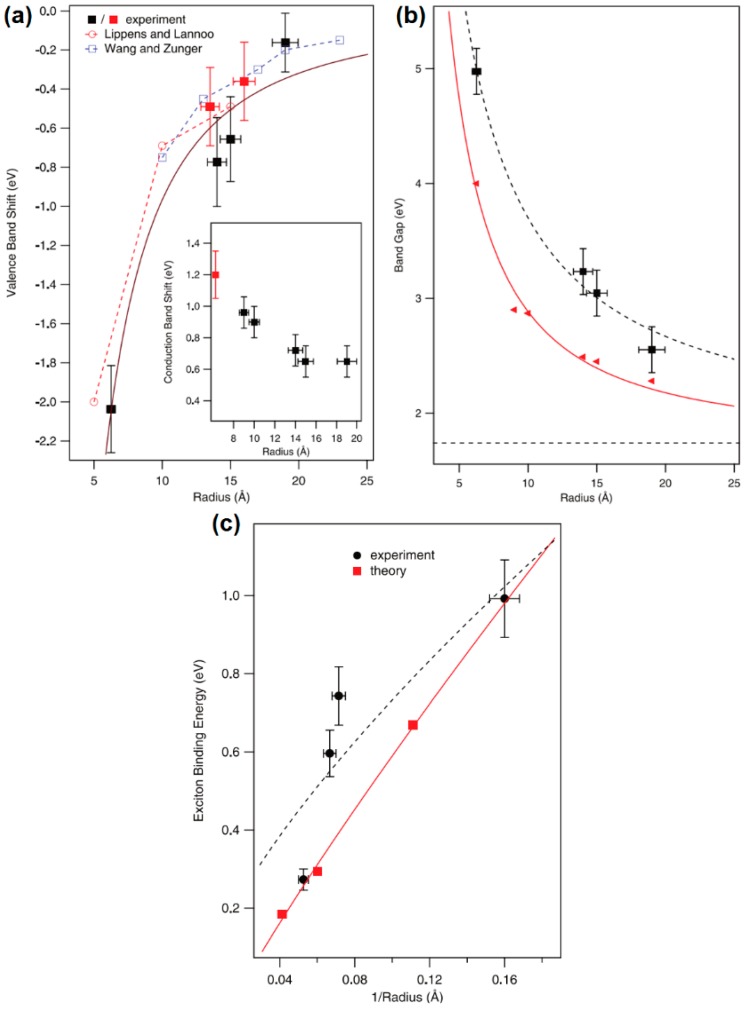
(**a**) Variation in the positions of the valence band and (inset) conduction band extrema as a function of particle radius in CdSe QDs. The black and red filled squares represent the data points for pyridine and thioglycolic acid-functionalized CdSe QDs, respectively, while the open circles and squares represent theoretical values. (**b**) Effect of the CdSe QD particle radius on the electronic bandgap. The black presents the CdSe QD photoemission bandgaps while the red represents the CdSe QD optical absorption band gaps. (**c**) Exciton binding energy of CdSe QDs as a function of particle radius. The black dots represent the CdSe-TOPO QD experimental exciton binding energy while the red squares represent the theoretical values. Adapted with permission from [42]. Copyright 2009 American Chemical Society.

In a BHJ solar cell, the electron generation usually happens in the donor material, as is the case in Figure 4, where the donor material is a conjugated polymer. When illuminated, excitons are generated. However, according to Strein et al. and Rosanna et al. and several other reports [34,38,39,40], the exciton can be generated at the donor side or the acceptor side or on both sides simultaneously. There is strong evidence for charge generation involving both donor and acceptor based on the action spectra plotted on the same graph as absorbance spectra as in Figure 5, where the combined efficiency becomes higher instead of lower as indicated in previous reports [40]. The close correspondence in the insets of Figure 5a–c between the external quantum yields in the near-infrared spectra range (where the conjugated polymers used do not absorb) and the absorption of the PbS QDs provides definitive proof of the contribution of the QDs to charge generation in the hybrid solar cell. In semiconducting polymers, the Frenkel exciton generated by photoexcitation can be decomposed into two effective particles; a relative particle consisting of a positronium atom with a screened electron–hole interaction that describes the size and binding energy of the electron–hole pair, and a centre-of-mass particle that describes the extent of delocalization of the electron-hole pair [41]. Thus, a unique situation arises wherein the wave function of the relative particle is highly localized onto a single monomer unit as it is tightly bound, while the wave function of the centre-of-mass particle is delocalized along the polymer chain over several monomer units. In II–VI and IV–VI semiconductor quantum dots (as in other inorganic semiconductors), Mott–Wannier excitons are generated, whose binding energies and energy levels are a function of the size of the quantum dots as shown in Figure 6 [42]. The monotonic increase in bandgap and exciton binding energy with decreasing QD size is shown in Figure 6b,c. Figure 6a shows that the valence band edge of CdSe QDs does not change appreciably for quantum dots up down to a size of 2.5 nm, but then can be tuned over a range of nearly 2.2 eV with decreasing QD size to 0.5 nm. The inset of Figure 6a indicates that a substantial shift of >0.6 eV in the position of the conduction band edge occurs even for 2.5 nm-sized CdSe QDs, but subsequent shifts are limited to another 0.6 eV as the QD size decreases to 0.5 nm. Therefore, in the 0.5–2.5 nm size range of QDs, shifts in the valence band maximum account for the major part of the increased bandgap while for larger quantum dots, shifts in the conduction band (CB) minimum are almost exclusively responsible for increases in the bandgap compared to the bulk. These results have practical utility; for instance, if a significant contribution of the CdSe QDs in the photogeneration of charge is desired in a hybrid solar cell, quantum dots of sizes >1.5 nm must be used. To a first approximation, if a higher driving force (proportional to the polymer LUMO-QD CB difference in Figure 3) is needed for the dissociation of Frenkel excitons generated in the polymer, larger CdSe QDs with lower conduction band edges with respect to the polymer LUMO can be used, but if a higher *V*_oc_ is desired—related to the polymer HOMO-QD VB difference in Figure 4—smaller QDs are preferable.

### 2.4. Charge Separation

**Figure 7:**
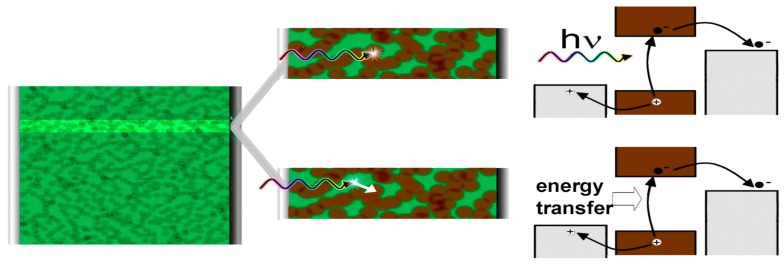
Schematic of a PSOTT/quantum dot blend “Schottky-diode” type junction. The hole-collecting electrode is denoted by a vertical light gray bar at left. The green is the PSOTT polymer and the brown circles are PbS quantum dots. The electron collecting LiF/Al contact is drawn as a darker grey vertical strip at right side. The highlighted horizontal strip shows two routes for generating photocurrent in the Schottky–diode device. In the upper image, a photon is absorbed by a quantum dot. In the bottom image, the photon is absorbed by the polymer and the energy of photon is then transferred to the quantum dot. The mechanisms drawn at the right simulates the dissociation of exciton by the mentioned mechanism. Reproduced from [34] with permission of the Royal Society of Chemistry.

**Figure 8:**
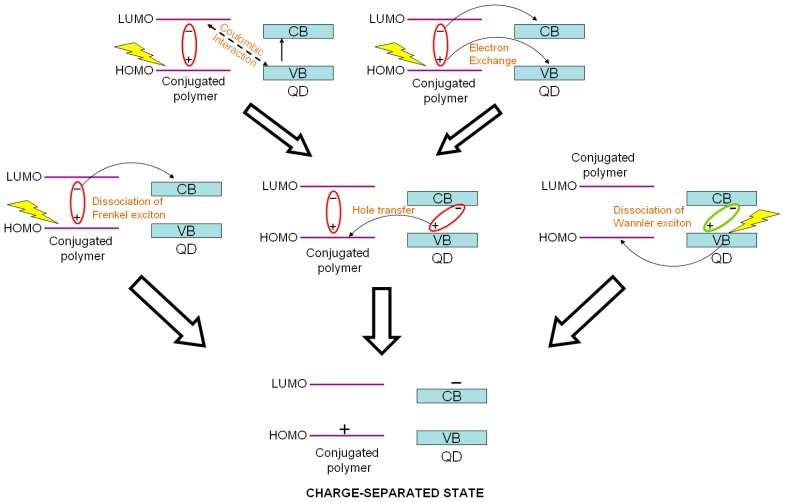
Charge separation mechanisms in polymer–quantum dot bulk heterojunction solar cells. Reprinted from [45] with permission from Elsevier.

As stated above, the exciton can be generated both in the polymer and QDs, but the separation of the as-generated exciton has several mechanisms. The conventional way is for the Frenkel exciton generated in the conjugated polymer (donor) to diffuse to the heterojunction interface, followed by exciton dissociation accompanied by electron transfer to the acceptor. There are two other reported mechanisms, expressed in the Figure 7. In quantum dot-conjugated polymer heterojunctions, the Wannier exciton generated in the quantum dot can be dissociated at the hetero-interface concomitant with hole transfer to the donor. Instead of dissociation of the Frenkel exciton, the energy stored in the exciton can be transferred to the quantum dots by energy transfer leading to another excitation, followed by dissociation and charge transfer as shown in Figure 7 [34]. Marcus-type electron transfer and/or hole transfer between the donor and acceptor at the interface are short range phenomena, which is why they occur only very close (~1 nm) to the hetero-interface. Non-radiative electronic excitation transfer can be either long-range (Förster-type) or extremely short-range (Dexter-type) in nature. Förster-type resonance energy transfer (FRET) is an orientation-dependent Coulombic interaction between the donor and acceptor transition dipole moments that varies inversely with the sixth power of the inter-chromophoric distance and which is strongest when there is a large overlap between the emission spectrum of the exciton donor and the absorption spectrum of the acceptor [43]. Dexter-type energy transfer occurs through an exchange interaction and has a biexponential dependence on inter-chromophoric distance [44]. The various charge separation mechanisms are schematically illustrated in Figure 8.

The mechanisms mentioned above are typically determined by steady state (SSPL) and time-resolved photoluminescence spectroscopy (TRPL) as well as by transient absorption spectroscopy (TA), also known as femtosecond/ultrafast pump-probe spectroscopy. In blends of PCPDTBT with *tert*-butylthiol capped CdSe QDs, Couderc et al. [46] demonstrated ultrafast electron transfer from the photoexcited CP to the quantum dots occurring over a time scale smaller than 100 fs. Lutich et al. [47] studied charge separation in equimolar blends of thioglycolic acid capped CdTe QDs (average size ~2.7 nm) and the conjugated polymer poly[9,9-bis(3′-((*N*,*N*-dimethyl)-*N*-ethylammonium)-propyl)-2,7-fluorene-*alt*-1,4-phenylene] dibromide (PDFD). An electrostatic interaction between the negatively charged side chains of PDFD and the positive charged surface ligands of the CdTe QDs promoted a conjugation between the polymer and the QDs. The PDFD-CdTe blend forms a type II heterojunction and there also exists excellent spectral overlap between the emissions spectrum of PDFD and the absorption spectrum of the CdTe QDs; thus, both electron transfer and energy transfer (see Figure 8) are possible pathways for the dissociation of the Frenkel exciton photogenerated in PDFD. Using SSPL and TRPL together with modelling of recombination kinetics, Lutich et al. [47] showed that energy transfer dominates—accounting for 70% of the dissociated excitons—and that, furthermore, the energy transfer was found to be nearly exclusively of the Förster-type rather than of the Dexter-type. Zhang and Xu synthesized small PbS QDs of slightly different sizes, directly in the polymer poly[2-methoxy-5-(2′-ethyl-hexyloxy)-*p*-phenylene vinylene] (MEH:PPV). They subsequently studied Dexter-type energy transfer from MEH:PPV to the PbS QDs upon exciting the polymer at 420 nm by monitoring the subsequent PL emission of MEH:PPV. An emission peak for the MEH:PPV-PbS QD blend was found for a QD diameter of 2.65 nm, which was attributed to slower Dexter energy transfer due to an extremum (minimum in this case) in the overlap between the emission spectrum of the polymer and the 1*S*_e_−1*S*_h_ and 1*P*_e_−1*S*_h_ transitions of the QDs [48].

### 2.5. Charge Collection

**Figure 9:**
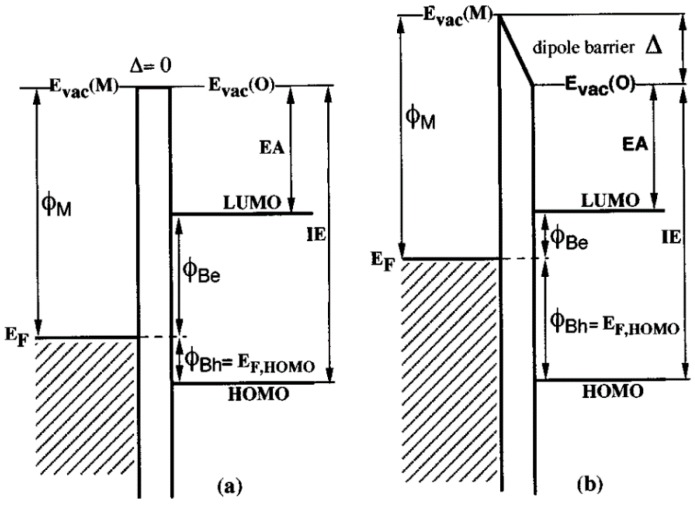
Energy level diagram of a metal–organic semiconductor interface (**a**) without an interface dipole **Δ** and (**b**) with the interface dipole. ϕ_M_ is the work function of the metal, *E*_F_ is the Fermi level, EA and IE are the electron affinity and ionization potential of the organic semiconductor, respectively; ϕ_Be_ and ϕ_Bh_ are the electron and hole barriers, respectively; HOMO and LUMO are the highest occupied molecular orbital and lowest unoccupied molecular orbital of the organic semiconductor, respectively; and E_vac_(O) is the vacuum level in the organic semiconductor, while E_vac_(M) is the metal vacuum level. Reproduced with permission from Ref. [49]. Copyright 1998 AIP Publishing LLC.

**Figure 10:**
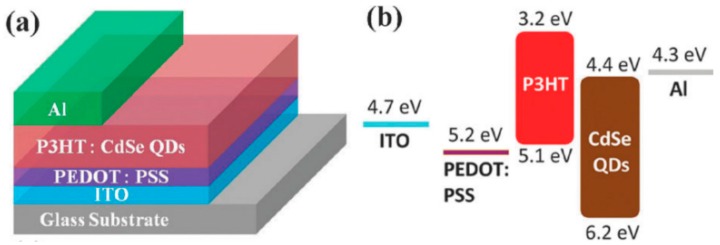
(**a**) A non-inverted structure of a BHJ solar cell composed of CP & QD; (**b**) the arrangement of energy levels of the materials used in the solar cell. Reproduced from [51] with permission of the Royal Society of Chemistry.

**Figure 11:**
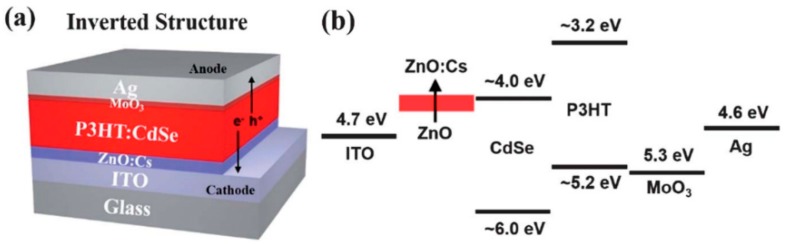
(**a**) An inverted structure of a BHJ solar cell composed of CP & QD blend; (**b**) the arrangement of energy levels of the materials used in the solar cell. Reproduced from [52] with permission of the Royal Society of Chemistry.

The positive and negative charges are collected separately at the anode and cathode, respectively, an energy level diagram of which is shown in Figure 3. The interface dipole that forms at organic semiconductor–electrode metal junctions (Figure 9) makes it challenging to form ohmic contacts to bulk heterojunctions by increasing the barrier to charge injection [49]. For instance, a layer of a high work function hole collector such as poly(3,4-ethylenedioxythiophene) polystyrene sulfonate (PEDOT:PSS) or MoO_3_ is nearly always required to create an ohmic contact to the conjugated polymer donor in order to construct a high efficiency hybrid solar cell. In non-inverted all-organic bulk heterojunctions (Figure 10), the electron-collecting contact typically requires a thin layer of an unstable low work function metal such as Ca, Mg or LiF followed by a layer of Al, which has a harmful effect on the ambient stability and operational lifetime of the resulting solar cell. On the other hand, in QD–polymer bulk heterojunctions where the QDs function as electron transporters, a thin film of Al is sufficient to form an ohmic contact with the QDs for optimal electron collection—a significant advantage. An ultrathin conjugated polyelectrolyte layer—such as cationic polythiophenes—has also been proposed as an electron collection layer in non-inverted solar cells with a modest amount of experimental justification [50]. There are also reports of an inverted structure, where a metal with a high work function such as Au or Ag is used to collect holes and a metal oxide layer with a low work function such as TiO_2_ or ZnO between the ITO and active layer is used to collect electrons. A typical inverted structure is shown in Figure 11.

## 3. Material Processing and Synthesis-Related Aspects

### 3.1. Conjugated Polymers

Conjugated polymers or conducting polymers, a discovery by Heeger (who was granted the Nobel Prize in Chemistry in 2000 for this discovery and subsequent work), opened a whole new field of material that is available for the application of optics and electronics [53]. Saturated polymers are all insulators and are of limited interest for electronics. On the other hand, conjugated polymers can be conducting or semiconducting, which makes them of significant interest for optoelectronic devices. Conjugated polymers have several advantages when compared to traditional inorganic semiconductors. To start with, single crystals and thin films of most inorganic semiconductors require high vacuum deposition, and/or energy- and cost-intensive elevated temperature processing to synthesize, while conjugated polymers can be produced from solution which is very cost- and energy-efficient. Moreover, they are flexible so that devices made from them can be flexible as well. They are also lightweight due to their low density, which makes transportation easier. Their properties such as the bandgap, HOMO/LUMO level positions, major carrier types (*n* or *p* type), and optical transparency are modifiable through engineering of their molecular structure. However, most work on the molecular engineering of polymers is still empirical due to an inadequate predictive understanding of the relationship between molecular structure and the physical and electronic properties of the polymer [54]. The synthetic complexity of many conjugated polymers and the questionable scalability of the synthetic processes are also factors to consider for eventual mass production and commercialization. Technical limitations include the relatively low carrier mobility (typically 10^−1^–10^−4^ cm^2^·V^−1^·s^−1^, high exciton binding energies and small exciton diffusion length (<20 nm) in comparison to conventional inorganic semiconductors and halide perovskites (where mostly free carriers are generated) [55,56,57]. The development of the BHJ concept was motivated by the need to reduce the exciton migration distances in CP-based solar cells.

Some common CPs used in BHJ solar cells are described in Table 1. As the synthesis methods of CPs are not the focus of this article, we refer readers elsewhere for the methods used to synthesize them [37,58,59,60].

### 3.2. Quantum Dots

Quantum dot nanocrystals have been the subject of intense study over the past two decades [61]. Quantum dots are typically made from binary compounds such as PbS, PbSe, CdS, CdSe, InAs, and InP, although ternary compounds are also possible. Their unique properties make them ideal for a number of applications, including photovoltaic devices. These nanocrystals are of such small size (smaller than the exciton Bohr radius) that quantum confinement occurs, and the properties deviate significantly from that of bulk materials [62]. The spectrum of possible energy states shifts from continuous to discrete and the band gap becomes wider as the particle becomes smaller. This effect becomes more pronounced the smaller the particle becomes, meaning that the band gap can be tuned to the desired size and quantum dots can be tailored to harvest different wavelength regions of the sun’s solar spectrum.

Another interesting property of quantum dots is the efficient multiple exciton generation (MEG) they demonstrate [63]. In conventional photovoltaic theory, incident photons produce a single exciton (electron hole pair). If the energy of the photon is greater than the band gap of the material, the excess energy is eventually lost as heat through the thermalization of so-called “hot carriers” [64]. If this excess energy is instead used to generate additional electron–hole pairs as in MEG, greater solar cell efficiencies can be obtained [65,66]. While MEG has been demonstrated in bulk materials with extremely high energy photons, only QDs have been able to demonstrate this process efficiently and with the lower energy photons that lie within the solar spectrum. The mechanism of MEG is of some debate [67]; however, it is usually attributed to the discrete energy state spectra and carrier confinement inherent in QDs. One difficulty associated with MEG is charge collection, as very fast charge separation times from QDs are required to outpace exciton–exciton annihilation and Auger recombination [68].

For almost any quantum dot application, it is desired to have a monodisperse, narrow size distribution to ensure consistent properties. Rapid nucleation followed by slow growth are the two steps typically required to obtain this size distribution; this is accomplished by solution-based method separating the nucleation and growth stages, the most common being the hot injection method where a room temperature precursor is injected into a surfactant at high temperature followed by reaction cooling [69]. The high concentration of coordinating ligands at elevated temperature ensure homogeneous nucleation while lowering the reaction temperature precludes overcoming the activation barrier for nucleation, thus allowing only crystal growth. The size of the resultant QDs can be tuned by controlling the temperature and reaction time.

**Figure 12:**
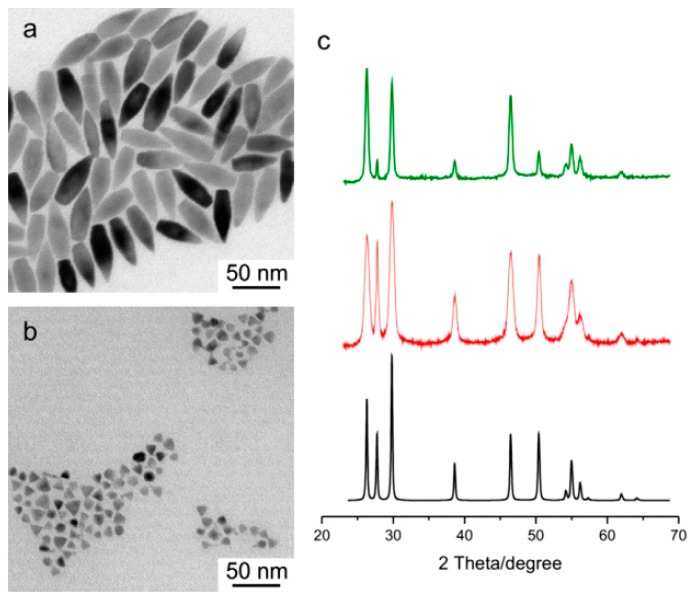
Colloidally synthesized CuInS_2_ quantum dots made by Radychev et al. for the purpose of hybrid solar cell blends with P3HT. (**a**) TEM images of pyramidal QDs; (**b**) TEM images of elongated QDs; (**c**) XRD diffraction patterns of elongated CuInS_2_ (green), pyramidal CuInS_2_ (red), and bulk CuInS_2_ (black). Reprinted from [70], with permission from Elsevier.

QDs may appear in many different shapes as well as sizes, a factor that can play a significant role in the performance of a resulting BHJ hybrid solar cell. They may be formed through additional processing steps. Structures such as nanorods, tetrapods and hyperbranched nanoparticles can enable the formation of a more well-defined nanocrystal-polymer interface in BHJ solar cells. An example of elongated and pyramidal CuInS_2_ nanoparticles as fabricated by Radychev et al. [70] is shown in Figure 12, where the resulting P3HT-blended hybrid solar cell was found to exhibit superior performance when using pyramidal as opposed to elongated QDs.

## 4. Design Considerations

### 4.1. Film Morphology

**Figure 13:**
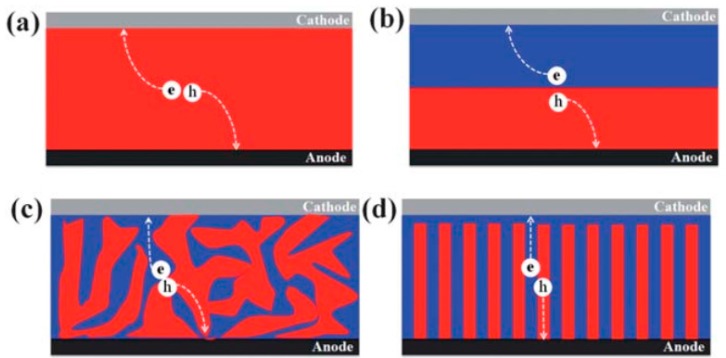
Schematic of various types of device architectures of solar cells. Red domains correspond to electron donor phases, while blue domains correspond to electron acceptor phases (**a**) Single-layer device, sandwiched between electrodes with different work functions; (**b**) bilayer device, with increased efficiency due to exciton dissociation occurring at the interface between phases; (**c**) bulk heterojunction device, where domain size is close to the exciton diffusion length of around 10 nm; (**d**) A proposed ideal film morphology, and ordered bulk heterojunction device where electrons and holes always have pathways to their respective electrodes. Reproduced from [72] with permission of the Royal Society of Chemistry.

In a BHJ solar cell, the device performance is heavily dependent on the morphology of the photoactive film layer, in this case the QD/conjugated polymer blend. In general, solar cells can be classified according to the architecture of their photoactive layers, as shown in Figure 13. Of greatest significance to this paper is Figure 13c, the BHJ solar cell. In this device configuration, the thoroughly mixed donor and acceptor domains result in a large interfacial area between the two. At the same time the domains are kept small—on the order of the exciton diffusion length at around 10 nm—so that excitons can diffuse to the donor/acceptor interface and can therefore be dissociated into electrons and holes.

**Figure 14:**
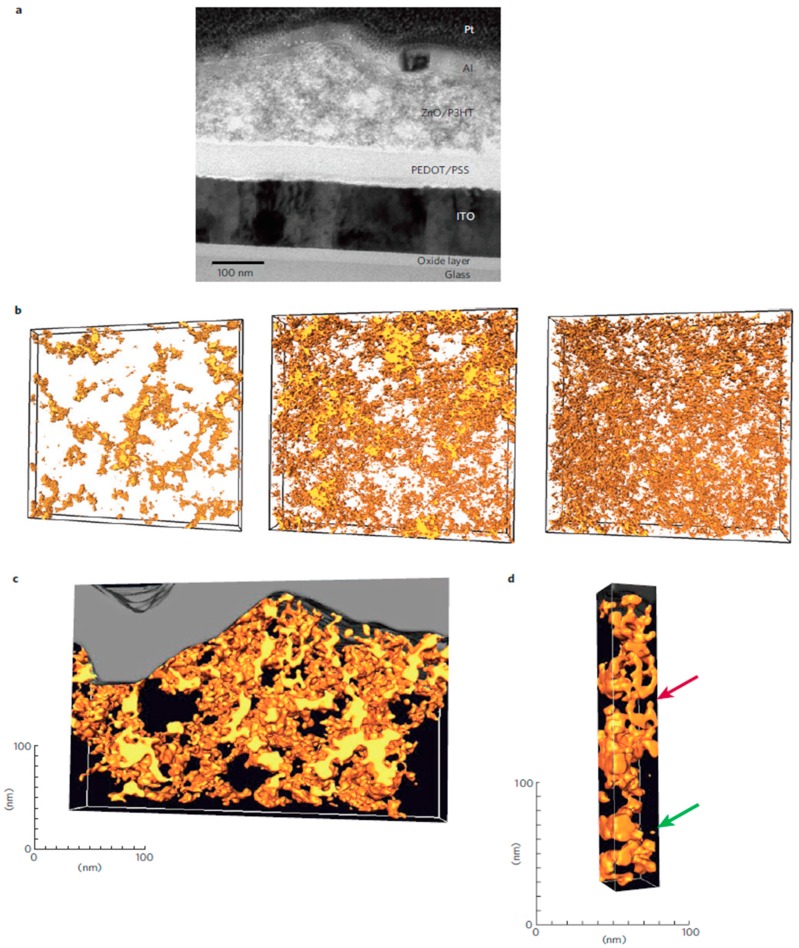
Electron tomography of ZnO/P3HT solar cells. (**a**) TEM image of a cross section of a ZnO/P3HT solar cell. (**b**) Reconstructed volumes of ZnO/P3HT layers. Three samples were analysed, with thicknesses of 57, 100, and 167 nm going from left to right. P3HT is transparent, while ZnO is yellow. (**c**) Reconstructed volume of a cross section of a ZnO/P3HT active layer in a complete device; (**d**) an image demonstrating ZnO domains connected to top but not flowing through to the bottom (red arrow) and an entirely isolated ZnO domain (green arrow). Reprinted with permission from Macmillan Publishers Ltd: Nature Materials [71], Copyright 2009.

Another aspect of morphology to consider involves ensuring that each domain is interpenetrated and forms a continuous pathway for the free electrons and holes to travel to their respective electrodes, preferably in a strictly vertical path (Figure 13d). If continuous pathways are not formed, then recombination will occur as the free electron or hole reaches a second donor/acceptor interface, resulting in charge losses. In practice, however, it is impossible to construct a photoactive layer with an ideal morphology as defects and imperfections occur. Oosterhaut et al. employed electron tomography to show the technical problems associated with achieving film morphologies in P3HT/ZnO solar cells that result in percolation pathways for both types of charge carriers, as shown in Figure 14 [71].

Film morphology is influenced by every device fabrication step associated with forming photoactive layers; this includes the materials and solvents used, the film thickness, the deposition method employed, and any post-fabrication procedures done such as annealing. The best choice of parameters is usually determined on a case-by-case basis, making optimization a difficult process. However, it is generally known that the miscibility of the donor and acceptor phases plays the largest role in the resulting film morphology, which is associated with the surface energy of the blending components [73]. Additives such as iodoalkanes and alkanedithiols have been successfully used to improve the morphology and the resulting conversion efficiency of methanofullerene-CP BHJ solar cells [74,75]. The differential solubility of the fullerene component in the additive, and a higher boiling point of the additive compared to the host solvent have been identified as key criteria for morphology-improving additives [76]. An improved phase separation with larger interconnected regions of the CP coupled with improved π-stacking, greater intercalation of the fullerene in the polymer and suppression of the fullerene aggregation are thought to be responsible for the positive effects of the additive on the morphology and photovoltaic conversion efficiency of the devices fabricated using the additives.

### 4.2. Material Selection

**Figure 15:**
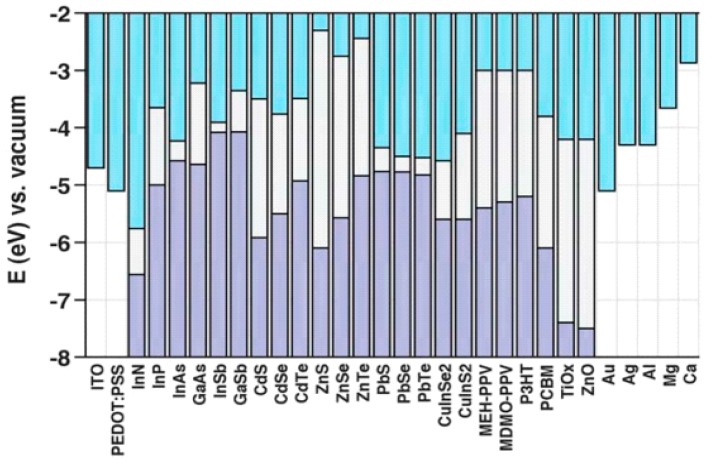
The energy levels of various materials used in hybrid solar cells, where the grey region corresponds to the electronic band gap present in such materials. Reproduced from [37] with permission of the Royal Society of Chemistry.

The QD and CP materials used in a BHJ solar cell are selected strategically; some QD materials work better with certain CPs and vice versa. The primary design considerations are the energy levels and band gaps of the materials to ensure efficient charge transport. When a CP is used as the donor material, it is desirable to have the LUMO band positioned above the QD acceptor conduction band so that photogenerated electrons can be injected from the donor to the acceptor. Also, electrode materials are chosen so as to minimize the energy barrier for charge injection and extraction [77]. The energy levels of common materials used in BHJ solar cells are shown in Figure 15. In semiconductor nanocrystals, the band gap can vary depending on the dimensions of the nanocrystal in question, while the energy gap between the HOMO and LUMO energy levels in polymers can depend on factors such as the chain regularity. Furthermore, energy levels may shift as materials are brought into contact with each other.

The electrode materials are also chosen to ensure charge selectivity, so that electrons will flow to the cathode and holes to the anode. Electron transporting layers (which block the flow of holes) and hole transporting layers (which block the flow of electrons) are used to increase charge selectivity. It is common to use wide bandgap metal oxides such as ZnO or TiO_x_ as the electron transporting layer as they are optically transparent with inherent *n*-type behavior [78]. Hole transporting layers are chosen to have a high work function, matching the high HOMO level of donor polymers and the work function of metal electrodes. The most widely used hole transporting layer is PEDOT:PSS, although it is highly hydrophilic and commonly produces adhesion problems when fabricating devices [79]. In comparison, compounds such as MoO_3_ or WO_3_ provide better stability and performance.

The desire for proper miscibility, phase segregation and good energy alignment between donor and acceptor materials has meant that considerable experimentation has occurred with different combinations of materials. Some of the most commonly investigated donor conjugated polymers include P3HT, P3DT, PCPDTBT, MEH-PPV, and MDMO-PPV. These compounds have been used with a wide variety of nanocrystal QDs, including those made of CdS, CdSe, PbS, and PbSe [40,55].

### 4.3. Device Architecture and Fabrication

The ordering of the material layers of a BHJ solar cell, and the methods by which they are formed plays an important role in device performance. As explained previously, the basic form of the device involves a photoactive layer sandwiched between two electrodes, often with an electron transport layer and a hole transport layer in between the photoactive layer and each electrode. Critical to the functioning of the device, however, is the ability of light to pass through one side of the device and reach the photoactive layer; thus, one side of the device must be transparent. The most common way of achieving this is to utilize a transparent conductive oxide (TCO) such indium tin oxide (ITO) or fluorine tin oxide (FTO) on a glass substrate as the anode. Examples of devices utilizing this device architecture are shown in Figure 10 and Figure 11, with the corresponding energy levels of the chosen materials shown as well. There has recently been a surge of interest in applying insights from nanophotonics to improve light absorption and optimize the distribution of photogenerated charge in bulk heterojunction solar cells.

A significant advantage of BHJ solar cells is that they may be fabricated cheaply and directly from solution. A typical process to fabricate such a solar cell would involve spin coating of PEDOT:PSS on ITO-coated glass and allowing the layer to dry. The CP and the QD would be blended together in a solvent by means of direct mixing or through in situ QD formation in CP followed by spin-coating on top of the PEDOT:PSS. The hole-blocking layer and metal electrode contacts on top of the photoactive layer may then be deposited using techniques such as evaporation.

### 4.4. Device Performance

The performance of solar cells is most commonly reported as power conversion efficiency (PCE), which is the fraction of incident light that is converted to electricity. The PCEs of hybrid BHJ solar cells have increased dramatically in the past decade and a half since Alivisatos et al. demonstrated such a device was possible in 2002. The device consisted of CdSe nanorods mixed with P3HT and achieved a PCE of 1.7% [55]. Further research has increased efficiencies, especially as control of film morphologies has improved and better materials have become available. Hybrid solar cells using cadmium chalcogenides were the superior performers for several years, achieving efficiencies of around 3%–4% in part due to the mature synthesis process and excellent shape control [80]. Lead chalcogenides, however, were unable to climb past efficiencies of 1% for many years due to poor heterojunctions between the QDs and the donor polymers; this was in part due to the small energy difference between valence band and HOMO energy levels. Experimentation with different low-bandgap polymers such as PDTPBT allowed lead chalcogenide-based solar cells to climb to 3.8% [81]. Further work with lead chalcogenide/PDTPBT hybrids resulted in the current record for hybrid BHJ solar cells at 5.5% by Liu et al. [30]. Alloyed PbS*_x_*Se_1−*x*_ QDs were used as the acceptor materials, previously shown to have an improved *I*_sc_ and *V*_oc_ [82]. Furthermore, a unique film morphology was realized in the BHJ, by ensuring a polymer-rich region at the bottom of the BHJ and a QD-rich region at the top. Such a structure provided efficient charge separation and transport, and reduced charge recombination.

Despite the advances made in recent years towards the improvement of hybrid BHJ solar cells, they still lag behind other solar cell devices. The maximum power conversion efficiency obtained by depleted heterojunction QD solar cells is 10.7% as of 2015 [83] while the maximum power conversion efficiency achieved by polymer solar cells was 10.6% as of 2015 [84].

## 5. Factors Limiting the Quantum Yield and Overall Power Conversion Efficiency in QD-Polymer Hybrid Solar Cells

### 5.1. Poor Interfacial Electronic Coupling Due to Stabilizing Ligands

Stabilizing ligands with long alkyl chains such as oleic acid and oleylamine are used in colloidal QD synthesis to solubilize the nanocrystals, control their size and protect them from undesirable chemical reactions. However when QD films are cast along with conjugated polymers to form a photovoltaic device, the stabilizing ligands—which are insulating—inhibit exciton dissociation and charge transport [85]. There has been a concerted move in the hybrid solar cell field to move to replace long chain ligands stabilizing CQDs by shorter chain and aryl-type ligands capable of effective π-overlap. Even so, electronic compatibility of the ligands with the polymer and the correct orientation of the ligands on the surface are key considerations. The electronic interactions of ligands with QDs are highly surface-site-dependent and not uniform over the entire surface of the nanocrystal. For instance, a single molecule of octadecanethiol was found to quench the luminescence of CdSe quantum dots by nearly 50% [86]. It is also highly challenging to distinguish between free and bound ligands on the surface of nanocrystals QDs, which in turn makes it difficult to relate the surface functionalization to the electronic properties [87]. Nuclear magnetic resonance (NMR) spectroscopy is the gold standard to differentiate between free and bound ligands through changes in linewidth and chemical shifts, but it often requires high concentrations of the ligands and quantum dots rendering it insensitive to the first few binding events, which typically involve the most reactive sites on a QD specific to a particular ligand [87].

### 5.2. Local Crystallinity

If the crystallinity of either the donor, acceptor or both at a heterojunction interface is poor, there is a higher driving force needed for the dissociation of polaron pairs due to the lack of access to delocalized states promoting quick separation [88,89]. The local crystallinity of the polymer can also have non-negligible effects on the optical absorption through the effect of crystallinity on the conjugation length [89].

### 5.3. Traps

Dangling bonds at the surface of quantum dots, and impurities and imperfections in the bulk (core) of the crystal function as trapping sites for charge carriers. Deep traps, whose energy levels of the traps are several kT away from the respective band-edges, have a highly negative effect on the process of charge collection in hybrid solar cells because deeply trapped charged carriers either cannot be extracted or have very long extraction times, due to which the probability of recombination with oppositely charged carriers becomes dramatically enhanced. A higher surface to volume ratio corresponds to a higher density of trap states, and therefore smaller sized quantum dots are expected to have more traps than larger sized quantum dots. Indeed, Greenham and colleagues used a combination of transient absorption spectroscopy (TA), photo-induced absorption (PIA), light intensity dependence of *J*_sc_ and *V*_oc_, and transient photoconductivity measurements in CdSe QD-P3HT blend hybrid solar cells with three different average QD sizes (3.3, 4.4 and 5.3 nm) to deduce that traps were a major and much more significant source of losses in hybrid solar cells as compared with fullerene-CP bulk heterojunction solar cells. The above mentioned study by Greenham et al. [90] used butylamine-capped CdSe QDs and demonstrated a much higher solar cell performance with larger sized QD nanocrystals compared to smaller sized QDs. On the other hand, Ginger et al. [20] observed the opposite effect in hybrid solar cells containing 3-mercaptopropionic acid (MPA)-capped PbS QdDs blended with the conjugated polymer PTB1 wherein blends containing the smaller sized QDs significantly outperformed those with larger-sized QDs. These contrasting results once again underscore the importance of the capping ligand, and its effectiveness in passivating surface traps.

### 5.4. Achieving a Bicontinuous Percolation Network in Blends of QDs with Conjugated Polymers

As mentioned previously, a quantum dot-conjugated polymer blend needs to have percolation pathways for both electrons and holes in order to avoid efficiency losses in the resulting bulk heterojunction solar cell due to poor charge collection. Such percolation pathways are, however, difficult to engineer and Section 6.2 provides a thorough and in-depth overview of the different approaches used to achieve such pathways for both electrons and holes.

In addition, relatively well-balanced carrier mobilities for holes and electrons are needed in order to prevent the build-up of charge and the reduction of the fill-factor of solar cells due to space-charge limited currents [91].

## 6. Proposed and Realized Solutions to the Factors Limiting Performance in QD–Polymer Hybrid Solar Cells

### 6.1. Interfacial Engineering

Capping ligands on the surface of QDs can play an important role in their electronic properties. These ligands maintain colloidal stability in the solvents often used for processing and play an important role in the resulting shape and size distribution during synthesis of the QDs. They can also passivate charge trap sites, modify the photoluminescence intensity, and shift the energy levels of the QDs [92,93]. Recombination kinetics of QD–CP blends exhibit stretched exponential decays as opposed to the power law decays exhibited by fullerene–CP blends, and back-electron transfer from the QDs to the CPs (a loss mechanism) has been found to be highly sensitive to the type of organic ligands on the surface of the QDs [94].

One problem with ligands involves the increased spacing between QDs, which can mean lowered electronic charge transport [93]. Some strategies for dealing with this include substituting longer-chain ligands for shorter ones or using post-plasma treatment to eliminate the need for ligands entirely, although this can result in oxide compounds on the surface which can also obstruct charge transport [95]. Finally, ligands can drastically impact the resultant film morphology of a hybrid BHJ solar cell [96]. Ligand exchange of oleate-capped QDs with pyridine improved the charge transfer and charge transport characteristics of QD–polymer blends. Celik et al. [97] and Jeltsch et al. [98] used a mixture of pyridine-capped CdSe QDs and NRs blended with conjugated polymers to achieve hybrid solar cells with conversion efficiencies of 3.5%–3.6%. Infrared spectroscopic characterization of neat films of oleate-capped PbS following post-deposition treatment with different short chain ligands such as 1,2-ethanedithiol (EDT), 3-mercaptopropionic acid (MPA), malonic acid (MA) and tetrabutylammonium iodide (TBAI), showed a dramatic decrease in the intensity of the symmetric and anti-symmetric stretching modes of the CH_2_ group, indicating effective removal of the oleate ligands [99]. Furthermore, the MPA post-deposition treatment was found to result in the longest carrier lifetimes (lowest recombination) in PbS QD-PTB1 hybrid solar cells, resulting in high *V*_oc_ and *FF* [99]. At the same time, it is worth keeping in mind that studies of PbS QD-based depleted heterojunction solar cells have shown that Fermi-level pinning due to a significant density of deep-level hole trap states is a key limitation, and that short chain ligands (EDT, MPA) produce incomplete passivation of the surface of the QDs [100]. Iodide ligands have been shown to much more effective in passivating PbS QDs in depleted heterojunction solar cells. Brutchey et al. [23] exploited this concept in hybrid solar cells to obtain an efficiency of 4.8% in hybrid solar cells consisting of PbI_2_-passivated PbS QDs blended with the donor polymer poly[2,6-(4,4′-bis(2-ethylhexyl)dithieno[3,2-b:2′3′-d]silole)-*alt*-4,7-(2,1,3-benzothiadiazole)] (Si-PCPDTBT).

The effect of ligand type, ligand adsorption geometry, ligand length, surface inorganic shells and wide bandgap metal oxide scaffolds on energy levels and charge transfer processes in quantum dots have been summarized by Vokhmintcev et al. [101] in a recent review article. Vokhmintcev et al. [101] also discussed the much-ignored phenomenon of hole trapping in detail, pointing to the beneficial effects of correct surface stoichiometry and surface halogenation in suppressing hole trapping in II–VI and IV–VI QDs. Boles et al. [102] provided an excellent overview of the classification of QD capping agents into L-, X- and Z-type ligands, and their corresponding effects on QD energetics and charge transfer. A particularly useful aspect of the aforementioned review article [102] is the outlining of application-driven ligand design. The consequences of varying the organic ligands and the concentration of the nanocrystal precursors on the shape and aggregation of II–VI and IV–VI semiconductors were reviewed by Su et al. [103]. The engineering of intimate contacts between QDs and CPs to facilitate charge transfer through (i) one-step ligand exchange of insulating ligand capped QDs with functionalized CPs (ii) ligand exchange followed by direct covalent coupling of CPs with QDs (iii) direct grafting of CPs onto the surfaces of QDs and (iv) formation of nanocomposite blends through direct synthesis of QDs in a CP matrix, has been comprehensively reviewed by Zhao et al. [104].

### 6.2. Achieving a Bicontinuous Percolation Network in Blends of QDs with Conjugated Polymers

**Figure 16:**
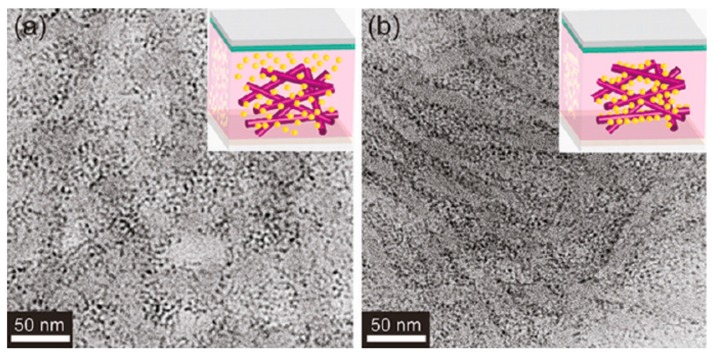
TEM images of P3HT/CdS QD hybrid films synthesized (**a**) without grafting and (**b**) using grafting process by solvent exchange. The inset images are schematic representations of each; CdS QDs are yellow spheres and P3HT NWs are purple lines. Reprinted with permission from [80]. Copyright 2011 American Chemical Society.

One approach to achieving bicontinuous charge percolation networks for electrons and holes is to make films from conjugated nanowires decorated by II–VI or IV–VI semiconductor QDs bound to the nanowires instead of merely solution casting blended solutions of polymers and dispersed QDs. The advantages of this approach are the higher charge carrier mobility of the polymer nanowire coupled with the more directional hopping transport of carriers along the quantum dots strung along the nanowires (see Figure 16). Xu et al. [105] formed P3HT–CdSe QD hybrid coaxial nanowires wherein the QDs were bound to the polymer nanowires (NWs) by non-covalent interactions, and observed a significantly higher photoconductivity as well as enhanced Frenkel exciton dissociation efficiency for films containing the hybrid coaxial NWs versus the blended composite. Likewise, Ren et al. [80] used an active layer consisting of CdS QDs bound to P3HT NWs through solvent-assisted grafting and ligand exchange, and achieved a solar cell conversion efficiency of 4.1%. A similar strategy was adopted by Chen et al. [106], who used CdTe nanorods or tetrapods as the acceptor in conjunction with a monoaniline end-capped conjugated polymer poly[(4,4′-bis(2-ethylhexyl)-dithieno[3,2-b:2′,3′-d]silole)-2,6-diyl-*alt*-(2,1,3-benzothiadiazole)-4,7-diyl] (PSBTBT-NH_2_) as the donor. The anchoring of CdTe QDs to the monoaniline endgroup of the polymer together with a benzenedithiol solvent vapor anneal improved charge transport as well as charge separation. An optimized 80:20 blend of CdTe QDs and PSBTBT-NH_2_ exhibited a η of 3.20%, double the performance of a similar optimized blend of CdTe QDs and PSBTBT (η = 1.59%) [106].

**Figure 17:**
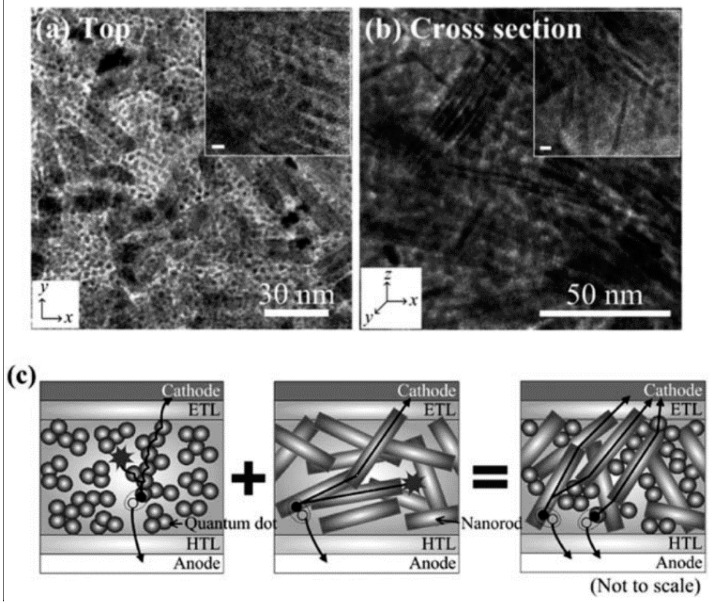
TEM images of the hybrid blend with QDs and NRs (0.3:0.7 wt/wt) in the PSBTBT matrix: (**a**) top and (**b**) cross-sectional views. The scale bars in the insets are 5 nm. Continuous interfacial contacts by both QDs and NRs are clearly shown in both horizontal and perpendicular directions of the film. (**c**) Cross-sectional schematic diagram of the three differently composed devices to explain the superiority of the hetero-structured blends. Reprinted with permission from Ref. [107]. Copyright Royal Society of Chemistry 2013.

In a hybrid solar cell based on a polymer–QD active layer, it is easier to obtain percolating paths for holes through the conjugated polymer (typically the donor) from cathode to anode due to the chain-like nature of the polymer and their plasticity, enabling them to form highly tortuous paths around the quantum dots. Therefore, the real problem in hybrid solar cells w.r.t. charge collection relates to the formation of continuous networks through the quantum dots for electron transport. Apart from forming polymer NWs and then decorating them with QDs, two strategies have been attempted to solve this problem, namely (i) the use of colloidal II–VI and IV–VI semiconductor nanorods to lengthen electron transport within the quantum dot phase (Figure 17) and (ii) the increasing of the quantum dot concentration vs. the concentration of the conjugated polymer. Both approaches have been used to achieve efficiencies of ca. 5% and constitute the current state of the art in non-perovskite hybrid solar technology. The potential of high aspect ratio chalcogenide nanorods (NRs) to provide directional charge transport pathways in hybrid solar cells was noticed by Huynh et al. [55], who used 7 nm × 60 nm CdSe NRs blended with P3HT in the active layer to obtain solar cell power conversion efficiencies of 1.7%. Using 4.4 nm × 32 nm CdSe NRs (aspect ratio of 7) blended with the polymer poly[2,6-(4,4-bis(2-ethylhexyl)-4H-cyclopenta [2,1-b;3,4-*b*]-dithiophene)-*alt*-4,7-(2,1,3-benzothiadiazole)] (PCPDTBT) in a 10:1 ratio by weight, and subjected to an 1,2-ethanedithiol (EDT) immersion treatment for 1 min following spin-coating, Zhou et al. [108] achieved an efficiency of 4.7% for their hybrid solar cells. Nam et al. [107] used a mixture of PbS_0.7_Se_0.3_ NRs and QDs blended with the polymer poly[2,6-(4,4′-bis-(2-ethylhexyl)dithieno [3,2-*b*:2′,3′-d]silole)-*alt*-4,7(2,1,3-benzothiadiazole)] (PSBTBT), subjected to an EDT immersion treatment after spin-coating, and obtained a solar cell power conversion efficiency of 3.4%.

**Figure 18:**
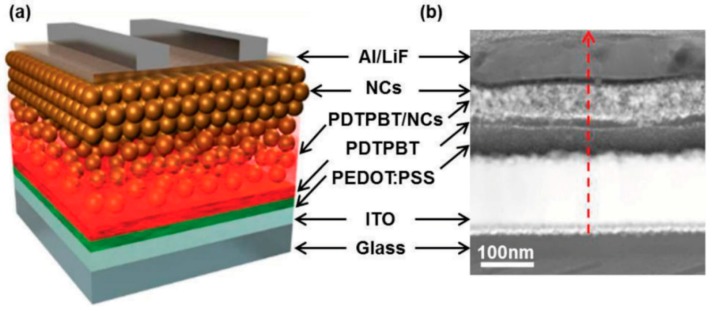
(**a**) Schematic illustration of a hybrid solar cell with a vertical D-D:A-A structure in the active layer of the device. The self-assembled vertical phase separation occurs in the polymer/QD blend film during spin-coating. Then pure NC layers are deposited on top of the blend. Reprinted with permission from John Wiley and Sons: Advanced Materials Ref. [30], Copyright 2013.

**Figure 19:**
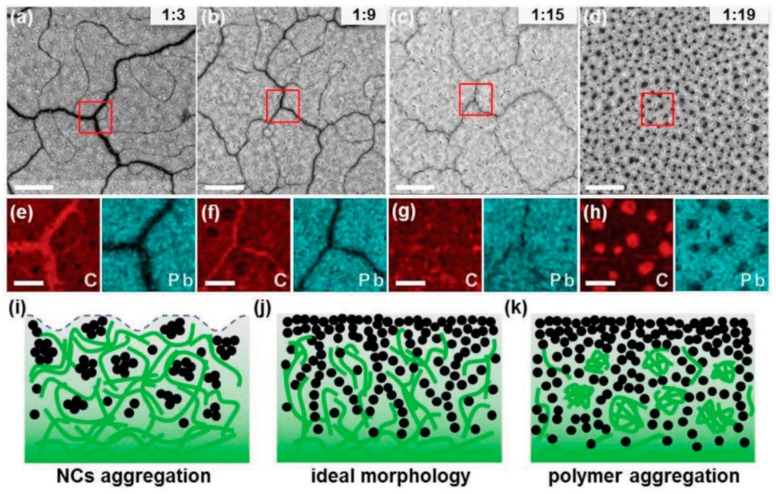
(**a**–**d**) Drift corrected HAADF-STEM images and (**e**,**f**) elemental mappings of C and Pb for PDTPBT/PbS_0.4_Se_0.6_ blend films (before the deposition of pure NCs) with various weight ratios: (**a**,**e**) 1:3; (**b**,**f**) 1:9; (**c**,**g**) 1:15; (**d**,**h**) 1:19. The scale bars for (**a**–**d**) represent 2 μm, (**e**–**h**) represent 500 nm. (**i**–**k**) Schematic depiction of the film morphology with increased NCs loading. (**i**) Low NC loading leads to the aggregation of NCs and incomplete coverage of NCs on bottom polymer. (**j**) Moderate blend ratio results in ideal film morphology. (**k**) Excess NC loading leads to the aggregation of polymers in NC matrix. Reprinted with permission from John Wiley and Sons: Advanced Materials Ref. [30], Copyright 2013.

Yao et al. [109] deployed a high concentration of II–VI semiconductor QDs in the active layer to obtain good electron percolation paths and used a polymerizable precursor to form the conjugated polymer donor around the QDs. Blended solutions containing high CdTe QD:PPV precursor ratios by weight (18:1 and higher, as high as 1:36) were spin-coated and then annealed at temperatures of 250 °C to form the active layers of bulk heterojunction-based hybrid solar cell devices that achieved conversion efficiencies of 3.6%–4.8% [29,109]. Mobility measurements using the space-charge-limited-current (SCLC) method indicated μ_e_ values of 1.6–3.2 × 10^−4^ cm^2^·V^−1^s^−1^, which are much higher than the electron mobilities of ~10^−5^–10^−6^ cm^2^·V^−1^·s^−1^ typically observed in II–VI semiconductor QD-conjugated polymer bulk heterojunctions [109]. Furthermore, the electron mobilities were relatively well-balanced with the hole mobilities. These carrier mobility results, together with *J*_sc_ values as high as 16 mAcm^−2^ confirm the efficacy of the strategy of increasing the quantum dot concentration (even after accounting for the higher density of the quantum dots) at the expense of the concentration of the conjugated polymer, leading to a device architecture that approaches a depleted heterojunction solar cell (see Figure 2b) wherein conversion efficiencies as high 10.6% have been obtained [110]. Liu et al. used a donor-(donor:acceptor)-acceptor (D-D:A-A) structure and an optimized blend ratio of the conjugated polymer poly(2,6-(*N*(1-octylnonyl)dithieno[3,2-b:20,30-d]pyrrole)-*alt*-4,7-(2,1,3-benzothiadiazole)) (PDTPBT) and PbSxSe alloyed QDs, respectively, to generate spontaneous vertical phase segregation as shown in Figure 18 [30]. The phase segregated structure (confirmed by a combination of AFM and HAADF-STEM) resulted in good percolation networks for both electrons and holes (Figure 19), and enabled the achievement of a solar cell conversion efficiency as high as 5.5% [30].

## 7. Conclusions

The operating principles of hybrid bulk heterojunction solar cells based on nanocomposites of II–VI and IV–VI inorganic semiconductor nanocrystal quantum dots with conjugated polymers, and the technical factors responsible for their non-optimal photovoltaic performance have been thoroughly elucidated. The two most important issues requiring further optimization—through materials improvements, clever device design and superior materials processing—are charge transfer and charge transport. Methods to enhance both have been presented at length. It is clear that hybrid BHJ solar cells are far from being viable for commercial application given that their efficiency lags behind that of other technologies. Issues such as unbalanced carrier mobilities and the need for electronically transparent ligands that simultaneously passivate traps and control nanocrystal size mean that considerable improvements are required. Also critical to improving hybrid BHJ solar cells is a better control over film morphologies to achieve near-unity charge collection within the BHJ blend. While the specific role of ligands in charge trapping and charge separation processes are still not completely clear at the present, the general rule is that II–VI and IV–VI inorganic nanocrystal quantum dots capped by either short-chain ligands or no ligands at all tend to have a higher photovoltaic performance in blends with conjugated polymers.

Furthermore, even if hybrid BHJ solar cells were successful enough to move from the lab to real-world applications, there are significant concerns about the ability to reproduce film morphologies at larger scales. Notwithstanding these concerns, even if hybrid BHJ solar cells are never able to overtake other photovoltaic technologies in terms of efficiency, they are an important topic of study with respect to the fundamental working mechanisms of solar cells, such as charge separation and transport. A further understanding of these mechanisms, and of the materials that comprise BHJ solar cells, will help in the further development of photovoltaics.

## Figures and Tables

**Table 1:** Common CPs used in BHJ solar cells.

Abbreviation	Name	Structure
P3HT	Poly(3-hexylthiophene)	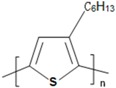
PCPDTBT	Poly[2,6-(4,4-bis-(2-ethyhexyl)-4*H*-cyclopenta[2,1-b;3,4-b′]-dithiophene)-*alt*-4,7-(2,1,3 benzothiadiazole)]	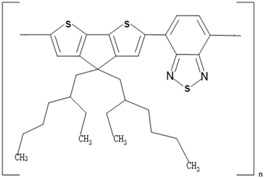
MEH:PPV	Poly[2-methoxy, 5-(2′-ethyl-hexyloxy)-*p*-phenylenevinylene)]	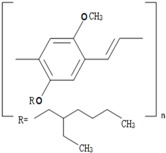
P3DT	Poly(3-decylthiophene-2,5-diyl)	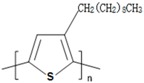
MDMO-PPV	Poly[2-methoxy-5-(3′,7′-dimethyloctyloxy)-1,4-phenylenevinylene]	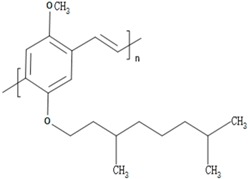
PDTPBT	Poly(2,6-(*N*-(1-octylnonyl)dithieno[3,2-*b*:20,30-*d*]pyrrole)-*alt*-4,7-(2,1,3-benzothiadiazole))	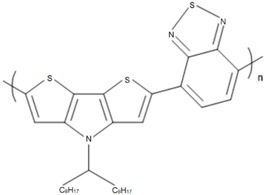
PTB1	Poly(4,8-bis (octyloxy) benzo (1,2-b:4,5-b′ dithiophene-2,6-diyl) (2-((dodecyloxy) carbonyl) thieno(3,4-b) thiophenediyl))	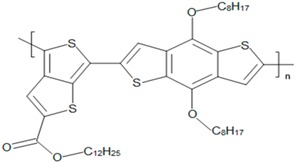
PSBTBT	Poly[(4,4′-bis(2-ethylhexyl)-dithieno[3,2-*b*:2′,3′-*d*]silole)-2,6-diyl-*alt*-(2,1,3-benzothiadiazole)-4,7-diyl]	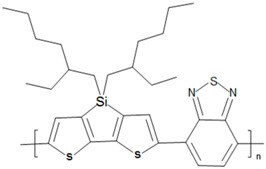
PPV	Poly(*p*-phenylene vinylene)	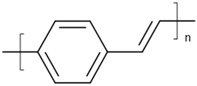
PDFD	Poly[9,9-bis(3′-((*N*,*N*-dimethyl)-*N*-ethylammonium)-propyl)-2,7-fluorene-*alt*-1,4-phenylene] dibromide